# The malignant dialogue between cancer-associated fibroblasts and osteosarcoma cells: microenvironment-mediated drug resistance and therapeutic targets

**DOI:** 10.3389/fimmu.2025.1621521

**Published:** 2025-08-20

**Authors:** Xiufeng Wang, Cong Luo, Danying Zhang

**Affiliations:** ^1^ Department of Orthopedic Trauma, Zhuji People’s Hospital of Zhejiang Province, Shaoxing, China; ^2^ Department of Emergency and Critical Care, Shanghai Changzheng Hospital, Naval Medical University, Shanghai, China

**Keywords:** osteosarcoma, cancer-associated fibroblasts, chemoresistance, extracellular matrix, tumor microenvironment

## Abstract

Cancer-associated fibroblasts (CAFs) are pivotal in shaping the immunosuppressive and chemoresistant tumor microenvironment (TME) of osteosarcoma (OS). This review explores how CAFs drive OS progression through paracrine signaling (e.g., TGF-β, IL-6), extracellular matrix (ECM) remodeling, exosome-mediated crosstalk, and metabolic reprogramming. We highlight CAF heterogeneity (e.g., myCAFs, iCAFs) and their roles in therapy resistance, emphasizing emerging strategies such as FAP inhibitors, TGF-β blockers, and CXCR4 antagonists. Combining these approaches with immunotherapy or chemotherapy offers promise for overcoming chemoresistance. Challenges like CAF plasticity and biomarker development are discussed, alongside future directions for precision targeting in OS.

## 1 Introduction

Osteosarcoma (OS) is a highly aggressive and easily metastasizing malignant bone tumor originating from mesenchymal cells in the bone marrow cavity or bone surface (1, 2). OS predominantly affects children and adolescents, with a peak incidence between 10 and 19 years of age (3). This age correlation stems from rapid skeletal growth during this period, where increased bone cell proliferation and differentiation elevate the risk of malignant transformation (4). OS cells exhibit marked cellular atypia and possess the unique ability to directly produce osteoid matrix or immature bone tissue, which serves as the pathological hallmark for diagnosis (5, 6). The treatment of OS typically employs multi-agent chemotherapy regimens to enhance therapeutic efficacy. Standard chemotherapeutic agents include methotrexate, cisplatin, doxorubicin and so on (7). However, the clinical utility of these drugs is significantly constrained by the development of chemoresistance and severe adverse effects (8–10). Indeed, overcoming drug resistance remains a key challenge in OS research.

The tumor microenvironment (TME) refers to the local milieu surrounding tumor cells, encompassing not only the malignant cells themselves but also adjacent stromal cells, extracellular matrix (ECM), cytokines, chemokines and metabolic byproducts (11–13). Cancer-associated fibroblasts (CAFs) represent a predominant cellular component of the TME, exhibiting remarkable functional and molecular heterogeneity (14). Through the secretion of cytokines, chemokines, growth factors and extracellular matrix components, CAFs significantly promote tumor progression and confer treatment resistance (15). For example, periostin, a CAF-secreted protein, promoted platinum drug resistance in ovarian cancer cells through activation of the PI3K/Akt signaling pathway (16). More importantly, a recent study demonstrated that CAFs promoted the occurrence of OS through the MIF-CD74 signalling axis, and their abundance was strongly correlated with the prognosis of OS patients (17). Recent single-cell RNA sequencing studies have revealed that CAFs are not a uniform population but rather consist of multiple functionally distinct subtypes that differentially influence OS progression and therapy resistance (18). This heterogeneity manifests through diverse secretory profiles, metabolic programs, and interactions with tumor cells, which collectively shape the immunosuppressive and chemoresistant TME (19). For instance, inflammatory CAFs (iCAFs) and myofibroblastic CAFs (myCAFs) exhibit opposing roles in OS metastasis, with the former promoting immune evasion via IL-6/STAT3 signaling and the latter driving ECM remodeling to impede drug delivery (20). Understanding these subsets is critical for developing precision therapies targeting CAF-specific vulnerabilities. Therefore, targeting CAFs has emerged as a promising strategy for OS treatment (21).

In this review, we comprehensively analyze the contributions of CAFs to OS chemoresistance, elucidating the underlying molecular mechanisms by which CAFs regulate chemotherapy sensitivity in OS, thereby underscoring their potential as innovative therapeutic targets.

## 2 Communication mechanisms between CAFs and OS cells

**Figure 1:**
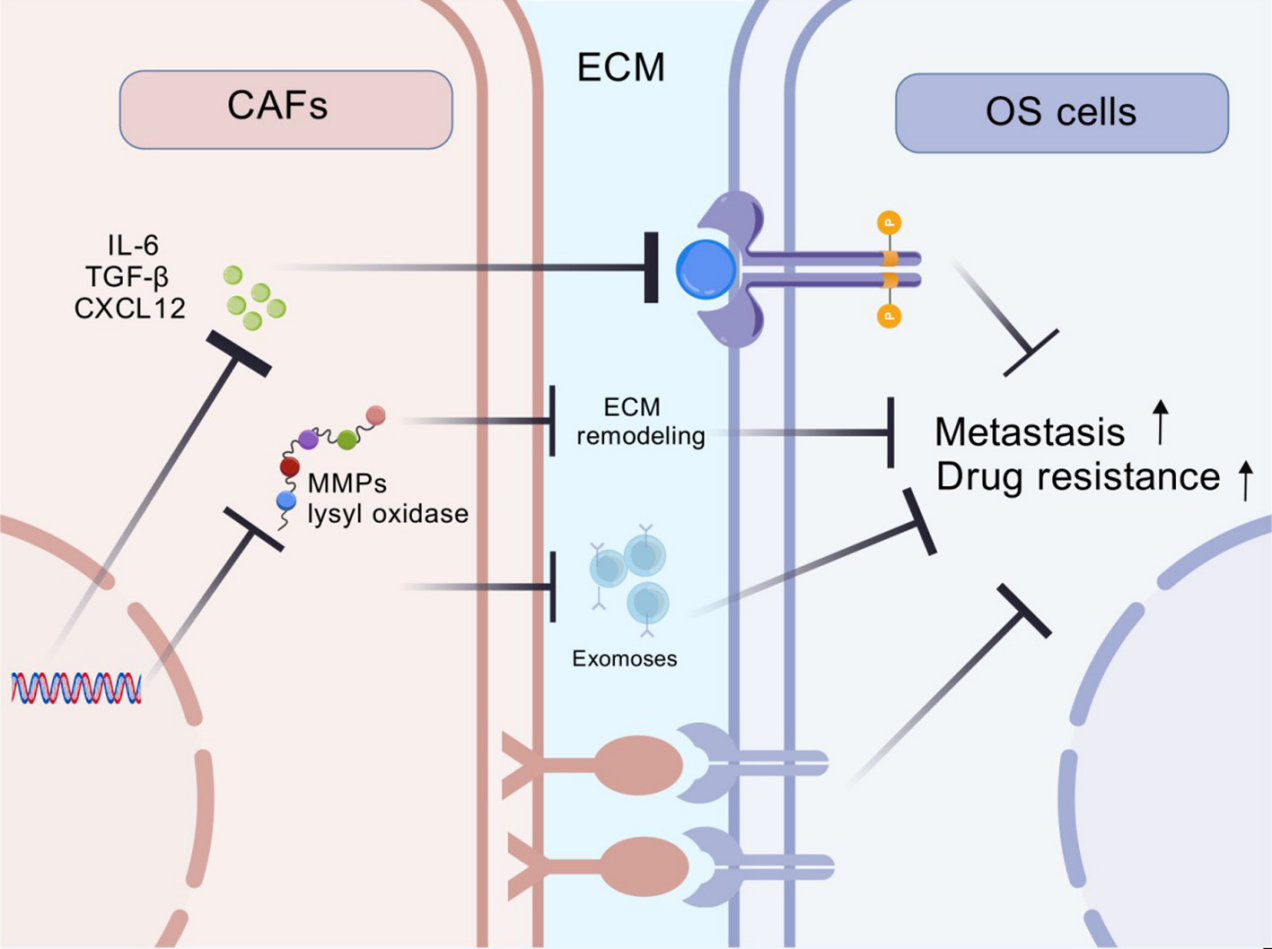
The key mechanisms of CAFs-mediated drug resistance and malignant progression in OS.

CAFs are a predominant component of the tumor microenvironment that modulate tumor cell proliferation, therapy resistance and immune evasion through diverse mechanisms. The interaction between CAFs and OS cells is summarized below (**Figure 1**).

### 2.1 Paracrine signaling

CAFs secrete diverse cytokines and chemokines that regulate OS progression, immune responses and drug resistance. These secretory factors not only shape the composition and function of immune cells within the tumor microenvironment but also profoundly influence malignant biological behaviors of cancer cells.

CAFs are one of the primary sources of TGF-β (22). Studies demonstrate that TGF-β influence cellular behavior through both SMAD-dependent and SMAD-independent pathways, inducing epithelial-mesenchymal transition (EMT) and thereby enhancing tumor cell migration and invasion (23). In OS, elevated TGF-β expression is closely associated with tumor metastasis and recurrence (24). TGF-β also plays a critical role in the development of tumor drug resistance. It enables a wide range of tumour cells to resist chemotherapy and radiotherapy by modulating different signalling pathways (25).

TGF-β derived from CAFs orchestrates a multifaceted resistance network: (1) Tumor-intrinsically, it activates SMAD4-dependent transcription of ABCB1 drug efflux pumps while suppressing pro-apoptotic Bim via HIF1α stabilization (26); (2) Immunologically, TGF-β polarizes macrophages to M2 phenotypes through SMAD3/IL-10 signaling and recruits Tregs via CCL22/CCR4 axis activation (27); (3) Stromally, it induces LOXL2-mediated collagen cross-linking that physically impedes drug penetration (28). This tripartite mechanism creates a chemoprotective niche, as demonstrated by a 68% reduction in cisplatin uptake when OS cells are co-cultured with TGF-β-secreting CAFs (24).

Furthermore, TGF-β establishes an immunosuppressive axis through: (1) Upregulating PD-L1 on CAFs via p38 MAPK/STAT3 signaling, enabling T-cell exhaustion through PD-1 ligation (29); (2) Inducing FAS ligand expression that triggers apoptosis of tumor-infiltrating CD8+ T cells (30); (3) Activating IDO1/kynurenine pathway in dendritic cells, which expands myeloid-derived suppressor cells (31). This network is clinically relevant, as OS patients with high TGF-β activity show 3.2-fold fewer infiltrating cytotoxic lymphocytes than low-TGF-β counterparts (32).

This comprehensive network of TGF-β-mediated effects underscores its central role in both immune evasion and chemotherapy resistance in osteosarcoma, highlighting the importance of targeting TGF-β signaling pathways in therapeutic strategies.

CAFs also secrete IL-6, which promotes tumor cells proliferation and invasion through multiple mechanisms (33). Studies showed that IL-6 activates the STAT3 signaling pathway, thereby enhancing OS cell proliferation and metastasis (34). In U2OS and MG-63 OS cells, IL-6 promoted cancer stemness and tumorigenicity by activating the OPN-STAT3 pathway (35). Besides, irisin reversed IL-6-induced EMT process in OS cells via the STAT3/Snail signaling pathway, consequently suppressing cancer cell migration and invasion (36). Additionally, the inhibition of IL-6 increased cisplatin resistance in human OS cells (37). In summary, IL-6 played a pivotal role in the initiation, progression, and drug resistance of OS.

CXCL12 is another important chemokine secreted by CAFs (38). The interaction between CXCL12 and its receptor CXCR4 promoted the proliferation and invasion of pancreatic cancer cells (39). In neuroblastoma, SOX17 inhibited cancer cell proliferation and invasion through the CXCL12/CXCR4 signaling axis (40). In OS, CXCL12 expression was epigenetically regulated. Studies demonstrated that CXCL12 expression was downregulated in OS cells via DNA methyltransferase 1, thereby regulating the metastasis and immune response in OS (41).

### 2.2 ECM remodeling

CAFs play a pivotal role in the TME of solid tumors such as OS. CAFs interact with tumor cells through ECM remodeling, influencing tumor growth, metastasis and therapeutic response (42). The ECM is a complex network composed of various macromolecules, including collagen, fibronectin, laminin, hyaluronic acid and others, which provide structural support and biochemical signals for cells (43). In the tumor microenvironment, CAFs are the primary producers and significantly alter composition and physical properties of ECM (44).

CAFs secrete enzymes such as lysyl oxidase to promote collagen cross-linking, thereby increasing ECM stiffness and density (28). These alterations not only provide a physical barrier for OS cells but also activate intracellular signaling pathways that enhance tumor cell invasion and metastasis (44). Additionally, CAFs produce matrix metalloproteinases (MMPs) to degrade ECM components, creating space for tumor cell migration (45). Meanwhile, CAFs regulate the deposition and organization of ECM constituents to guide directional tumor cell movement, further facilitating invasion and metastasis (42). Moreover, CAFs modify ECM architecture and mechanical properties through cellular contractile forces, influencing OS cells behavior (46).

### 2.3 Exosome-mediated communication

Exosomes are small extracellular vesicles secreted by cells that serve as critical mediators of intercellular communication between CAFs and tumor cells. CAFs utilize exosomes to transfer various bioactive molecules—including miRNAs, lncRNAs, proteins, and metabolites—to tumor cells (47). These molecules regulate gene expression in tumor cells, promoting proliferation, migration, and invasion (48, 49). CAF-derived exosomes induce EMT process, enhancing tumor cell metastatic potential (50). Exosomes derived from CAFs modulate other cells in the tumor microenvironment, such as immune cells and vascular endothelial cells, thereby promoting angiogenesis and facilitating tumor growth through inhibiting the activation of immune cells (51). In addition, exosomes produced by CAFs reduce tumor cell sensitivity to drugs and support tumor cell survival (50). Interestingly, tumor cells can also modulate CAFs properties through exosomes. For example, tumor-derived exosomes activate normal fibroblasts and induce the transformation from normal fibroblasts to CAFs, thereby further promoting tumor progression (52).

### 2.4 Direct cell-cell contact

The interaction between CAFs and OS cells includes direct cell-to-cell contact. Direct cell-to-cell contact facilitate membrane surface molecular engagements, such as receptor-ligand binding, which subsequently activate intracellular signaling pathways (53). This signal transduction critically influences OS cells proliferation, migration and invasive capabilities. The physical contact between CAFs and OS cells may induce cytoskeletal remodeling, thereby altering cellular morphology and motility. For instance, CAFs secrete small extracellular vesicles that mediate collagen cross-linking and promote EMT process through the p-FAK/p-paxillin/YAP signaling axis, ultimately enhancing OS cells invasion and metastasis (54). CAFs transfer metabolic substrates (lactate, pyruvate and ketone bodies) to OS cells via direct contact, supporting tumor cell growth and survival (55). This metabolic coupling enables tumor cells to better adapt to the nutrient-deprived and hypoxic conditions within the tumor microenvironment. Additionally, CAFs modulate immune cell function through direct contact. For example, CAFs directly suppresses T-cell activation through expressing immune checkpoint molecules PD-L1 (56).

### 2.5 CAFs heterogeneity in osteosarcoma

CAFs exhibit significant functional and molecular heterogeneity, which plays a crucial role in shaping the TME and influencing OS progression and therapy resistance. This heterogeneity arises from diverse cellular origins, spatial distribution within tumors, and dynamic interactions with other TME components, leading to distinct CAF subpopulations with varying pro-tumorigenic functions (57)

#### 2.5.1 Origins and subtypes of CAFs in OS

CAFs in OS can originate from multiple precursor cells, including resident fibroblasts, mesenchymal stem cells (MSCs), endothelial cells undergoing endothelial-to-mesenchymal transition (EndMT), and even transdifferentiated osteoblasts (58). Single-cell RNA sequencing studies have identified at least three major CAF subtypes in OS: Myofibroblastic CAFs (myCAFs): Characterized by high expression of α-SMA (ACTA2) and TGF-β signaling markers, these CAFs are typically located near tumor cells and contribute to extracellular matrix (ECM) remodeling and mechanical stiffness (59); Inflammatory CAFs (iCAFs): Enriched in cytokine secretion (e.g., IL-6, CXCL12) and JAK/STAT signaling, iCAFs promote immune suppression and angiogenesis (60);Antigen-presenting CAFs (apCAFs): Express MHC class II molecules and co-stimulatory proteins, potentially modulating T-cell responses (59).

#### 2.5.2 Functional implications of CAF heterogeneity

The spatial distribution of CAF subtypes correlates with distinct pathological features of OS. For example, myCAFs are predominantly found in the tumor core, where they drive collagen cross-linking and create a physical barrier to drug penetration, while iCAFs localize to the invasive front, facilitating metastasis through immune evasion (59). Metabolically, CAF subpopulations exhibit divergent behaviors. Lactate-secreting CAFs (marked by MCT4 overexpression) fuel OS cell glycolysis, while lipid-rich CAFs promote chemoresistance by transferring fatty acids to tumor cells via direct contact or exosomes (61). This metabolic coupling is further regulated by hypoxia, with peri-necrotic CAFs showing upregulated HIF-1α signaling and enhanced secretion of pro-angiogenic factors like VEGF (62). Single-cell studies identify COL11A1+ CAFs as a chemoresistance-driving subtype in OS, activating IGF-1R/Akt signaling to promote cancer stemness (42). Conversely, CD10+ CAFs recruit tumor-associated neutrophils (TANs) via CCL2 secretion, accelerating lung metastasis (63). These findings underscore the need for subtype-specific targeting, such as COL11A1-neutralizing antibodies.

CAFs directly modulate immune cell function through multiple mechanisms. For instance, PD-L1 overexpression on CAFs inhibits CD8+ T cell activation by binding to PD-1, facilitating immune evasion (56). Additionally, CAF-secreted IL-6 and TGF-β polarize macrophages toward an M2 phenotype, which further suppresses antitumor immunity (35). Single-cell RNA sequencing reveals that COL11A1+ CAFs correlate with T cell exhaustion markers in OS, suggesting subtype-specific immunosuppressive roles (57). Single-cell studies also identify COL11A1+ CAFs as a chemoresistance-driving subtype in OS, activating IGF-1R/Akt signaling to promote cancer stemness (58). Conversely, CD10+ CAFs recruit tumor-associated neutrophils (TANs) via CCL2 secretion, accelerating lung metastasis (18). These findings underscore the need for subtype-specific targeting, such as COL11A1-neutralizing antibodies.

## 3 Therapeutic strategies targeting CAFs to overcome OS resistance

**Figure 2:**
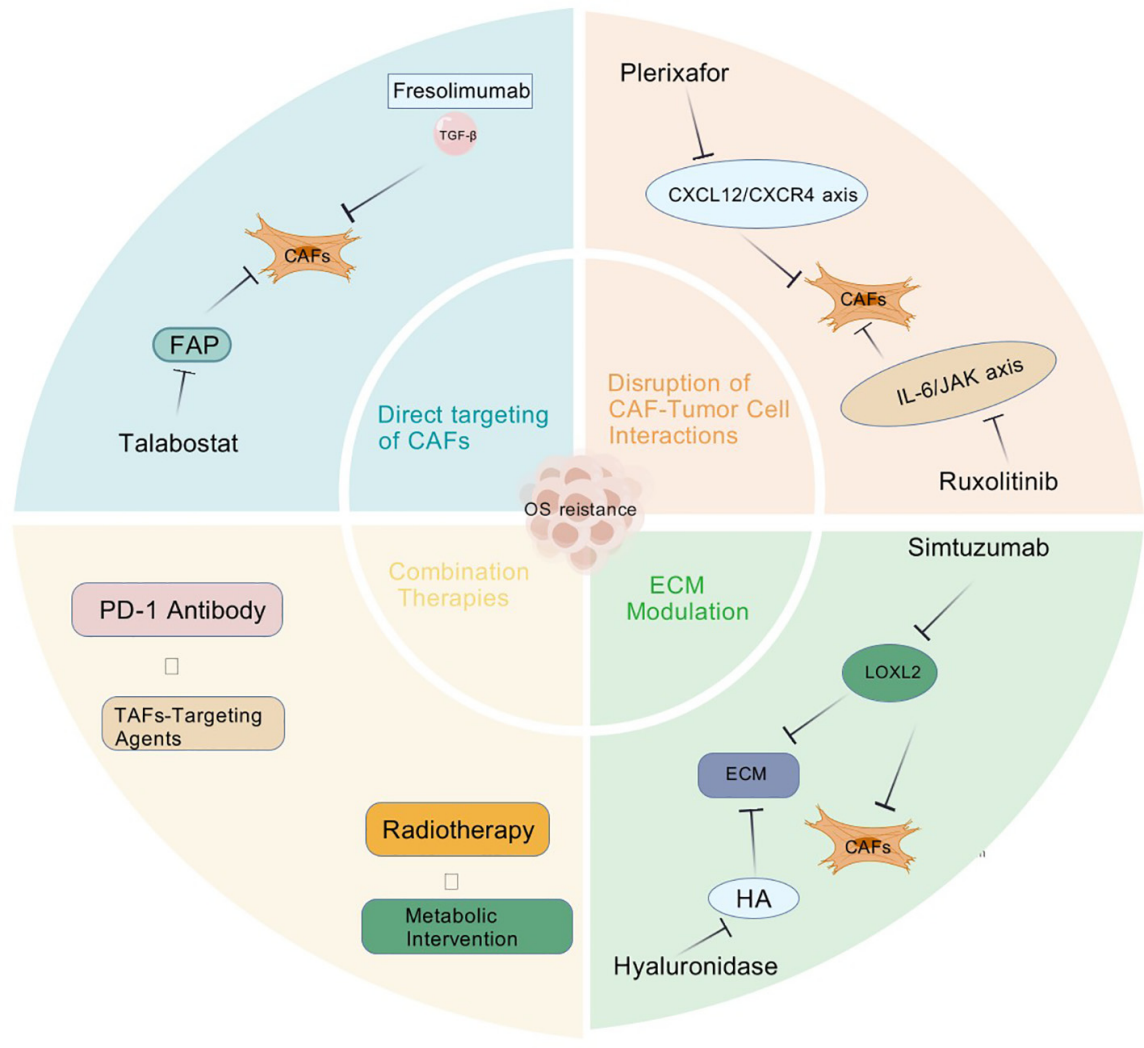
Several therapeutic strategies targeting CAFs to overcome OS resistance via: (1) Direct inhibition (FAP/TGF-β blockers); (2) Disrupting crosstalk (CXCR4/IL-6 inhibitors); (3) ECM modulation (LOXL2/hyaluronidase).

Targeting CAFs has emerged as a crucial strategy to overcome OS drug resistance. Below are the primary CAF-targeting approaches and their research advancements (**Figure 2**).

### 3.1 Direct targeting of CAFs

#### 3.1.1 Fibroblast activation protein inhibitors

FAP is a specific marker on the surface of CAFs, and its high expression in CAFs makes it an ideal target for targeting these cells (64). FAP inhibitors work by suppressing the activity of FAP, thereby reducing the pro-tumor effects of CAFs and enhancing the sensitivity of tumors to chemotherapy and immunotherapy (65). For example, studies have shown that Talabostat (an oral FAP inhibitor) significantly reduces the activity of CAFs, thereby inhibiting OS growth and invasion (66). Moreover, Talabostat exhibits synergistic effects when combined with other anticancer agents. A phase II clinical trial evaluated the antitumor activity of Talabostat in combination with pembrolizumab (an anti-PD-1 antibody) in patients with advanced solid tumors, and the results revealed that the development of tumor was notably suppressed (67). Recent phase I trials have shown promising safety and preliminary efficacy of FAP-targeted therapies in solid tumors. For example, FAP-2286, a novel radioligand therapy, demonstrated favorable tumor uptake in a phase I study (NCT04939610) for advanced solid tumors (68, 69). However, OS-specific clinical data remain limited, partly due to heterogeneous FAP expression across CAF subtypes in osteosarcoma, potentially leading to variable responses. Additionally, on-target/off-tumor effects in normal tissues (e.g., healing wounds, fibrotic lesions) necessitate careful monitoring. Ongoing trials combining FAP inhibitors with immune checkpoint blockade (e.g., NCT05552703) may offer insights for OS treatment strategies (68).

#### 3.1.2 TGF-β neutralizing antibodies

TGF-β is a multifunctional cytokine, playing a crucial role in various biological processes, including cell growth, differentiation, and immune regulation. In the context of cancer, TGF-β has a dual role: it can act as a tumor suppressor in the early stages of tumorigenesis but often promotes tumor progression, metastasis, and immune evasion in advanced stages (31). The TGF-β signaling pathway is a crucial regulator of CAF phenotype and function. Blocking TGF-β can inhibit the transformation of normal fibroblasts into CAFs, reduce the expression of markers such as α-SMA, and thereby diminish the tumor-promoting activity of CAFs (70). Additionally, CAFs promote collagen cross-linking and EMT process through the release of sEVs, and inhibiting TGF-β affects this process (54). CAFs can influence the recruitment and differentiation of immune cells through various pathways, thereby impacting tumor immune evasion. The inhibition of TGF-β can alter the immunomodulatory function of CAFs, promoting immune cell infiltration and antitumor immune responses (30). Fresolimumab is a humanized monoclonal antibody that exerts its therapeutic effects by specifically neutralizing TGF-β. It displays significant potential in modulating the TME and regulating CAFs activity (71). In mouse models of breast cancer and pancreatic cancer, Fresolimumab treatment significantly reduced CAF activation and decreased ECM deposition, thereby inhibiting the formation of physical barriers and promoting drug delivery and immune cell infiltration (72). Fresolimumab treatment also improved the immune microenvironment of the TME, increasing the infiltration of cytotoxic T cells and reducing the levels of immunosuppressive cells such as regulatory T cells and myeloid-derived suppressor cells, thereby alleviating immune suppression and enhancing the antitumor activity of cytotoxic T cells (73, 74). Although Fresolimumab has potential, it is important to note that TGF-β may play a dual role in different tumor types and disease stages. In some cases, TGF-β may suppress early tumor development, while in advanced stages, it may promote tumor progression. Therefore, the use of TGF-β inhibitors such as Fresolimumab requires careful evaluation and should be combined with personalized treatment based on the specific conditions of the patient. Fresolimumab, a TGF-β neutralizing antibody, has shown mixed results in clinical trials. As of September 2023, TGF-β neutralizing antibodies are under clinical investigation for osteosarcoma, often combined with immunotherapy. For example, TQB2858, an anti-PD-L1/TGF-β bispecific antibody, is being explored in clinical settings (75). Preclinical studies indicate that TGF-β promotes chemoresistance and tumor progression, prompting trials testing its blockade to overcome resistance. Early approaches, such as combining anti-TGF-β with dendritic cell therapy, show antitumor potential. Given the dismal <30% 5-year survival rate in metastatic osteosarcoma (76), targeting TGF-β—a key mediator of bone metastasis—represents a promising strategy to improve outcomes.

### 3.2 Disruption of CAFs-tumor cells interactions

CAFs play a critical role in tumor progression and drug resistance through their interactions with tumor cells. CAFs support tumor cell growth, invasion, and drug resistance via multiple mechanisms, including the secretion of cytokines, chemokines, growth factors, and ECM components. Therefore, disrupting CAF-tumor cell interactions has become an important strategy to overcome tumor drug resistance.

#### 3.2.1 CXCR4 antagonists

CXCR4 (C-X-C chemokine receptor type 4) is a G protein-coupled receptor (GPCR), and its ligand CXCL12 (also known as stromal cell-derived factor-1, SDF-1) plays important roles in various physiological and pathological processes. The CXCL12/CXCR4 axis is involved in multiple critical processes, including cell proliferation, survival, migration, invasion and metastasis, and is associated with more than 20 different types of cancer (77). Upon binding of CXCL12 to CXCR4, multiple downstream signaling pathways are activated, including G proteins, PI3K/AKT, MAPK and RhoA/ROCK2 pathways (78). After G protein activation, it further regulates adenylate cyclase, phospholipase C, and others, generating second messengers such as cAMP, IP3, and DAG, thereby influencing cellular functions (79). The CXCL12/CXCR4 axis also plays a crucial role in guiding cell migration, particularly in immune cell homing, hematopoietic stem cell mobilization and tumor metastasis (80). The high expression of CXCL12 in the bone marrow directs leukemia stem cells expressing CXCR4 to localize within the bone marrow microenvironment, maintaining LSC quiescence and protecting them from chemotherapy (80). Tumor cells, by expressing CXCR4, respond to CXCL12 secreted by metastatic target organs (such as lymph nodes, lungs, liver and bone marrow), thereby promoting directional migration of tumor cells (81). The CXCL12/CXCR4 axis is involved in various inflammatory and immune responses. In a rat model of vascular dementia, inhibition of the CXCL12/CXCR4 axis alleviates neuroinflammation and cognitive dysfunction (82). Plerixafor was initially developed as an anti-HIV drug and later identified as a potent CXCR4 antagonist, subsequently approved for hematopoietic stem cell mobilization in autologous stem cell transplantation (83). Currently, plerixafor has demonstrated promising antitumor effects in various tumor models, particularly in overcoming drug resistance, with applications in both hematologic malignancies and solid tumors (84). The mechanism of action of plerixafor primarily revolves around the inhibition of the CXCL12/CXCR4 axis, exerting multiple effects in cancer therapy (85). In preclinical models of breast cancer and pancreatic cancer, plerixafor combined with chemotherapeutic agents (such as paclitaxel) significantly reduces tumor burden and improves survival rates (86). Additionally, plerixafor has been explored for enhancing the efficacy of immunotherapy by improving immune cell infiltration and augmenting antitumor immune responses. In p53-related therapies, CXCR4 can serve as a target in combination with anti-PD1 therapy (87). Although plerixafor has shown potential in both preclinical and clinical studies, its application in cancer treatment still faces challenges. For instance, some studies indicate that in Ewing sarcoma cell lines, plerixafor may instead promote cell proliferation and activate receptor tyrosine kinase signaling (88). Overall, as a CXCR4 antagonist, plerixafor influences tumor cells and the tumor microenvironment through multifaceted mechanisms, holding broad clinical prospects in OS applications.

In p53-mutant OS, CXCR4 inhibition has emerged as a promising strategy to enhance immunotherapeutic responses, especially when combined with anti-PD1 therapy. This approach leverages the role of CXCR4 in shaping the TME and modulating immune cell infiltration. Inhibiting CXCR4 can reduce tumor growth and potentially restore or enhance the efficacy of immune checkpoint inhibitors. This strategy aligns with broader efforts to convert “cold” tumors into “hot” ones by enhancing tumor immunogenicity and improving responses to immune checkpoint blockade (ICB) (89). For instance, in pancreatic cancer, inhibiting tumor-associated neutrophils (TANs) enhances the effectiveness of anti-PD-1 therapy (90). Similar strategies could be applied to OS, considering the immunosuppressive features associated with p53 mutations. Mechanistically, p53 mutations often lead to immune evasion and resistance to apoptosis, which can be countered by strategies that induce ferroptosis or modulate immune cell infiltration (91, 92).

#### 3.2.2 IL-6/JAK inhibitors

IL-6 is a pleiotropic cytokine that plays a critical role in various physiological and pathological processes, particularly in the tumor microenvironment, where it promotes tumorigenesis and progression by activating the JAK/STAT3 signaling pathway (93). Upon activation of the IL-6/JAK/STAT3 signaling pathway, the expression of genes such as Cyclin D1 and Bcl-2 is upregulated, promoting tumor cell cycle progression and inhibiting apoptosis, thereby enhancing tumor cell proliferation and survival (94). Activated STAT3 can induce the expression of EMT-related transcription factors, including Snail, ZEB1, and Twist, downregulate E-cadherin expression, and upregulate N-cadherin expression, thereby promoting the EMT process in tumor cells and enhancing their invasive and metastatic capabilities (94, 95). The activation of the IL-6/STAT3 pathway is associated with resistance to multiple chemotherapeutic drugs. STAT3 activation reduce tumor cell sensitivity to chemotherapy by regulating the expression of drug transporters, enhancing DNA repair capacity, or influencing apoptosis pathways (96, 97). Ruxolitinib is a JAK1/2 inhibitor that exerts anti-inflammatory and anti-tumor effects by inhibiting the JAK-STAT signaling pathway. Numerous studies have demonstrated its potential therapeutic value in various cancers, as it can influence tumor cell biology through multiple mechanisms. In renal cell carcinoma, Ruxolitinib suppresses tumor cell proliferation and survival by inhibiting the IL-6/JAK/STAT signaling pathway and downregulating PIM1 expression (98). In head and neck squamous cell carcinoma, Ruxolitinib overcomes EGFR-TKI resistance by blocking IL-6/STAT3 signaling, thereby improving therapeutic efficacy (99). Similarly, in NSCLC cells, Ruxolitinib reverses cisplatin resistance by inhibiting the JAK/STAT pathway (100). Additionally, Ruxolitinib suppresses pancreatic cancer progression by attenuating the pro-tumor effects of tumor-associated macrophages through inhibition of the STAT3 signaling pathway (101). In glioma cells, Ruxolitinib exhibits a dose-dependent inhibitory effect on interferon γ-dependent JAK/STAT signaling, thereby impairing tumor cell invasion and tumorigenesis (102). Recent research has shown that targeting the IL-6/JAK/STAT3 signaling pathway can significantly inhibit osteosarcoma growth and metastasis by reducing tumor self-seeding and enhancing antitumor immunity (103). For instance, a study demonstrated that the STAT3 inhibitor cryptotanshinone effectively reduced tumor progression and improved survival rates in osteosarcoma models by inhibiting IL-6 signaling (103). Additionally, JAK inhibitors are being explored in combination with other therapies to enhance treatment efficacy, as seen in preclinical and early-phase clinical trials (104). These findings suggest that IL-6/JAK inhibitors hold promise for improving outcomes in osteosarcoma patients, although further clinical trials are needed to confirm their safety and efficacy (105).

### 3.3 ECM modulation

ECM is a critical component of the tumor microenvironment, providing structural support and regulating cellular behavior (106). Abnormal ECM remodeling can contribute to tumor progression, metastasis, and drug resistance. Modulating ECM components disrupt these supportive interactions and enhance the efficacy of cancer therapies.

#### 3.3.1 LOXL2 inhibitors

Lysyl oxidase-like 2 (LOXL2) is an enzyme that catalyzes the cross-linking of collagen fibers, contributing to the stiffness and rigidity of the ECM (107). Elevated LOXL2 activity in the tumor microenvironment can promote tumor cell invasion and resistance to therapy (108). Inhibiting LOXL2 can reduce ECM stiffness and disrupt the pro-tumor effects of CAFs. Simtuzumab is a humanized monoclonal antibody that specifically targets LOXL2. By binding to LOXL2, Simtuzumab inhibits its enzymatic activity, thereby reducing collagen cross-linking and ECM stiffness. Preclinical studies have shown that Simtuzumab can decrease tumor-associated fibrosis and improve the efficacy of chemotherapy and immunotherapy (109). In clinical trials, Simtuzumab has demonstrated promising results in reducing tumor stiffness and enhancing drug delivery to tumor cells (109).

#### 3.3.2 Hyaluronidase

Hyaluronic acid (HA) is a major component of the ECM, contributing to its viscoelastic properties and influencing cellular behavior. High levels of HA in the tumor microenvironment can create a dense and impenetrable ECM, limiting drug delivery and promoting tumor progression (108). Hyaluronidase is an enzyme that degrades HA, thereby reducing ECM density and enhancing drug penetration. Hyaluronidase can be administered systemically or locally to degrade HA in the tumor microenvironment. By reducing HA levels, hyaluronidase can enhance the permeability of the ECM, allowing better penetration of chemotherapy drugs and immune cells. Preclinical studies have shown that combining hyaluronidase with chemotherapy or immunotherapy can significantly improve treatment outcomes (110). For example, PEGPH20 (a pegylated form of hyaluronidase) has been shown to reduce tumor interstitial pressure and enhance drug delivery in various cancer models (111).

### 3.4 Combination strategies

Combination therapies are becoming increasingly important in the treatment of cancer, as they leverage the synergistic effects of multiple treatment modalities to enhance efficacy and overcome resistance mechanisms. By integrating different therapeutic approaches, combination therapies can target multiple pathways involved in tumor progression and resistance, leading to improved patient outcomes.

#### 3.4.1 Chemotherapy + immune checkpoint inhibitors

Combining chemotherapy with immune checkpoint inhibitors has emerged as a powerful strategy to enhance anti-tumor effects by leveraging the cytotoxic effects of chemotherapy and the immune-boosting effects of checkpoint inhibitors (112). Nivolumab is a humanized monoclonal antibody that targets the PD-1 receptor, thereby blocking the inhibitory signals that cancer cells use to evade the immune system (113). By combining Nivolumab with CAF-targeting agents, such as TGF-β inhibitors or FAP inhibitors, the therapy can simultaneously reduce the immunosuppressive effects of CAFs and enhance the immune response against tumor cells (114). Preclinical studies have shown that this combination can significantly increase the number of tumor-infiltrating lymphocytes (TILs) and improve overall survival rates in various cancer models (115).

#### 3.4.2 Metabolic intervention + radiotherapy

Metabolic interventions aim to disrupt the altered metabolism of cancer cells, making them more susceptible to other treatments. Combining metabolic interventions with radiotherapy can enhance the efficacy of radiation by targeting metabolic pathways that contribute to radioresistance. Monocarboxylate transporter 4 is involved in the transport of lactate and other monocarboxylates, contributing to the acidic tumor microenvironment and promoting radioresistance (116). AZD3965 is a selective MCT4 inhibitor that blocks the transport of lactate, thereby reducing the acidic environment and enhancing the sensitivity of tumor cells to radiation (117). Studies have shown that AZD3965 can significantly enhance the efficacy of radiotherapy by normalizing the tumor microenvironment and reducing hypoxia (118). This combination has demonstrated promising results in improving local control and survival rates in various cancer models, including those resistant to conventional radiotherapy.

## 4 Challenges and future perspectives

CAFs significantly contribute to drug resistance in OS, a highly aggressive bone malignancy. These cells foster tumor progression and resilience to treatment through diverse mechanisms, such as secreting cytokines, chemokines, and extracellular matrix components. However, targeting CAFs to surmount drug resistance in OS faces several challenges. The heterogeneity of CAFs, with their varied phenotypes and functions, complicates the identification of specific targets and the development of effective therapies. Moreover, CAFs’ complex interactions with other cells in the tumor microenvironment, including tumor cells, immune cells, and endothelial cells, require a comprehensive understanding of the involved signaling pathways and potential compensatory mechanisms. Additionally, resistance to CAF-targeting therapies can emerge through genetic mutations, epigenetic changes, and adaptive responses within the tumor microenvironment. The lack of well-defined biomarkers to predict response to CAF-targeting therapies further hampers the development and application of personalized treatment strategies.

Despite these challenges, the future outlook for targeting CAFs in osteosarcoma is promising. Developing targeted therapies that specifically inhibit CAFs’ pro-tumor effects, such as TGF-β inhibitors, FAP inhibitors, and CXCR4 antagonists, holds potential for overcoming drug resistance. These therapies can be used alone or in combination with existing treatments to enhance efficacy. Combining CAF-targeting agents with chemotherapy, immunotherapy, or targeted therapies may provide synergistic effects, overcome multiple resistance mechanisms and improving patient outcomes. Advances in genomics, proteomics, and imaging technologies offer the potential for personalized medicine approaches. Identifying patients who are most likely to benefit from CAF-targeting therapies based on their tumor and CAF characteristics can improve treatment outcomes and reduce unnecessary side effects. Given CAFs’ immunosuppressive effects, combining CAF-targeting therapies with immunomodulatory agents may enhance anti-tumor immune responses, potentially overcome immune evasion mechanisms and improve the efficacy of immunotherapy in OS.

Long-term preclinical and clinical studies are essential to understand the chronic effects of CAF-targeting therapies and to identify potential late-onset resistance mechanisms. Future research should focus on elucidating the molecular mechanisms of CAF heterogeneity, developing biomarkers for CAF-targeted therapies, investigating the role of CAFs in immune modulation, and optimizing combination therapies. Specifically, further studies are needed to uncover the molecular pathways that drive the differentiation and functional diversity of CAFs in OS. This could involve single-cell sequencing technologies to identify novel CAF subtypes and their unique signaling pathways. Identifying reliable biomarkers that predict response to CAF-targeted therapies is crucial, and this could involve exploring the expression of specific proteins, miRNAs, or metabolic signatures in CAFs that correlate with treatment response. Understanding how CAFs interact with immune cells in the TME and identifying strategies to enhance immune surveillance by targeting these interactions could lead to more effective combination therapies. Preclinical studies should focus on optimizing the sequence and timing of CAF-targeted therapies with other treatments, such as chemotherapy or immunotherapy, to maximize therapeutic efficacy and minimize resistance. Conducting translational studies that incorporate patient-derived models and clinical trials will be essential to validate the efficacy of CAF-targeted therapies in OS and to identify potential challenges in clinical application.

In summary, while targeting CAFs in OS presents significant challenges, ongoing research and future studies hold promise for developing novel therapeutic strategies that can overcome drug resistance and improve patient outcomes. A multidisciplinary approach that integrates basic research, translational studies, and clinical trials will be critical in advancing this field. By addressing the heterogeneity of CAFs, understanding their complex interactions, and developing personalized combination therapies, we can pave the way for more effective treatments for osteosarcoma patients.
